# How the Cochrane Collaboration Is Responding to the Asian Tsunami

**DOI:** 10.1371/journal.pmed.0020169

**Published:** 2005-06-28

**Authors:** Prathap Tharyan, Mike Clarke, Sally Green

## Abstract

Tharyan and colleagues describe why the Cochrane collaboration made its library of systematic reviews freely available to affected countries.

**Figure:**
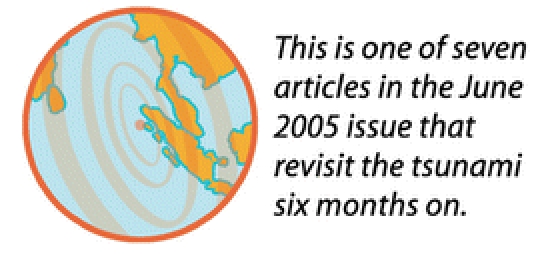


The powerful earthquake-triggered tsunami that devastated the coasts of many countries in two continents bordering the Indian Ocean on 26 December 2004 killed more than 280,000 people, displaced more than 1 million, and affected the lives of around 5 million more [1]. Unprecedented media coverage, in turn, triggered a worldwide outpouring of empathy, financial aid, and pledges of aid; the mobilisation of resources; and concerted action from governmental and nongovernmental organisations and international agencies such as the United Nations (Figure 1) and the World Health Organization. However, some of the well-meaning responses were not without drawbacks. There are concerns that the unregulated, uncoordinated, and poorly sustained activities of independent visiting health-care teams or individuals will undermine local health-care efforts [2].

**Figure 1:**
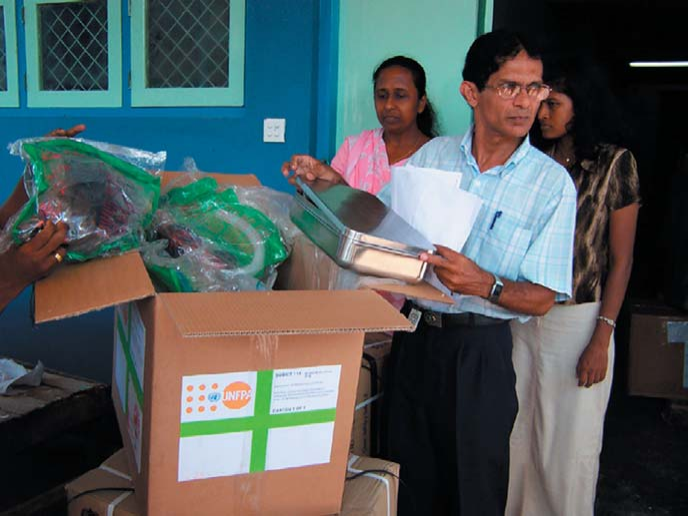
The United Nations Population Fund Sends Aid to Those Affected by the Tsunami Dr. Sanantha Wajewardena, the chief pharmacist at the Training Hospital in Galle, Sri Lanka, and two other pharmacists (background) unpack a shipment of essential safe-birthing supplies and surgical equipment supplied by the United Nations Population Fund to replace equipment that the hospital lost in the tsunami on 26 December 2004. (Photo: Joanne Ornag)

Six months after the tsunami, the attention of the media has largely shifted to other more pressing issues, leaving many unanswered questions about the appropriate response to such natural disasters. Is there a comprehensive list, prioritised and organised, of the health and social consequences of disasters? Do people have ready access to regularly updated evidence-based resources about the interventions relevant to such disasters? Is there a mechanism by which these resources could be made available to policy makers making decisions about the allocation of resources and interventions, as well as to people planning, providing, and receiving care in affected regions? Would these resources prove useful and, if so, which of the groups involved in disaster management should make use of them?

## The Need for Evidence-Based Interventions in Disaster Management

A commonly used strategy in the wake of traumatic events is brief “debriefing”, both voluntary and mandatory. The aim of debriefing is to reduce immediate psychological distress as well as to prevent the development of psychological disorders, notably post-traumatic stress disorder (PTSD).

In the wake of the tsunami, many teams rushed to the Nagapattinam district, one of the worst hit areas of Tamil Nadu, the state in India with the largest number of casualties from the tsunami. These teams offered forms of brief debriefing to survivors in each village before rushing on to the next of the 73 tsunami-affected villages in the district.

Prathap Tharyan was part of a team summoned by the government of Tamil Nadu to provide psychosocial support. The team checked the evidence and found a relevant Cochrane systematic review on the effects of debriefing [3]. The Cochrane review had not found evidence that brief single-session debriefing reduced psychological morbidity but showed, instead, that limited evidence from one trial indicated a significantly increased risk of PTSD at one year in those receiving debriefing (odds ratio 2.88 [95% confidence interval 1.11–7.53]) [3]. Because of this review, we urged officials and nongovernmental organisations to desist from offering brief, single-session debriefing.

This message about debriefing was incorporated into the content of counsellor training workshops along with evidence for interventions that were supported by the results of systematic reviews and randomised controlled trials [4–6]. Recent surveys of parts of the Nagapattinam district suggest that PTSD is not a significant mental-health problem among adult survivors of the tsunami.

Similarly, other evidence-based interventions, such as the distribution of insecticide-treated bed-nets [7], have helped in the prevention of outbreaks of malaria and dengue. Well-meaning but misdirected and sometimes harmful interventions could be prevented if those making decisions about the nature of responses had access to reliable and up-to-date evidence of what works and what does not.

## The Response of the Cochrane Collaboration

Shortly after the tsunami, it was felt that the Cochrane Collaboration, as the world's largest international organisation committed to providing good evidence about health care and with many members working in the region, had a moral duty to help in the global-relief and rehabilitation efforts. A working party was convened in early January 2005 of people in the region and elsewhere, with an E-mail discussion list and regular teleconferences aiding discussion and planning of initiatives. Further details, including a full list of the members of the working party, are available at http://www.cochrane.org/docs/asiancrisis.htm#response.

### Prioritisation of reviews of relevant health-care interventions.

A disaster of this magnitude raises the spectre of epidemics of infectious diseases and many other potential health-care problems. The working party, in consultation with all Cochrane entities and around 200 individuals from affected countries listed as contributors to the work of the Cochrane Collaboration, and members of other agencies such as the World Health Organization, Oxfam, and the publishers of BMJ's *Clinical Evidence*, drew up a list of over 200 interventions considered relevant to health care in the aftermath of the tsunami.

These topics were further prioritised and grouped to ascertain which interventions currently had an up-to-date Cochrane review and which would need Cochrane reviews to be updated or even commissioned. This list will be modified as further input from other sources becomes available, and it may become a valuable resource for coping with the aftermath of other disasters and health-care emergencies. The relevant reviews should provide a valuable one-stop resource for people making decisions about health care in the future.

### Disseminating the evidence.

The tsunami affected many countries where access to the Cochrane Library, which is available by individual or national subscription through Wiley InterScience (http://www.interscience.wiley.com/cochrane), is limited. The Cochrane Collaboration and John Wiley and Sons, the publishers, recognised the need to make Cochrane reviews more available and, so, agreed to provide free “one-click” access to all contents of the Cochrane Library for people in affected countries (Box 1) for a six-month period from February to July 2005 (http://www.thecochranelibrary.org). Governmental and nongovernmental agencies and institutions as well as individuals involved in health planning and health care in these countries now have access via the Internet to one of the best single sources of evidence on the effects of interventions likely to be useful in their efforts at no cost.

**Box 1. Countries Affected by the Tsunami That Qualified for Free Access to the Cochrane Library**

- Bangladesh
- India
- Indonesia
- Kenya
- The Maldives
- Malaysia
- Myanmar (Burma)
- The Seychelles
- Somalia
- Sri Lanka
- Tanzania
- Thailand

### Evidence aid: Summaries of evidence-based interventions.

Members of the working party, aided by others in the Cochrane Collaboration, are preparing concise evidence summaries of systematic reviews of topics of high priority. These summaries cover interventions relevant to infectious diseases, injuries and wounds, rebuilding of communities and infrastructures, mental health, nutrition, rehabilitation, and pregnancy and childbirth. They are available at http://www.cochrane.org/docs/tsunamiresponse. If a summary is not currently available but there is a relevant Cochrane review in the Cochrane Library, a link takes people straight to that review. If a suitable Cochrane review is not available, links are included to other identified sources of evidence, in particular, to topics in the BMJ's *Clinical Evidence* (http://clinicalevidence.com).

## Do We Know Enough to Deal Effectively with the Consequences of Disasters?

Sadly, the answer is “No, not nearly enough”. Of the topics in the list of the 200 or more interventions that are thought to be relevant to health care after a disaster such as the tsunami, there is an up-to-date, good-quality systematic review available for only a quarter of them. And, of these, not all have conclusions that can guide practice now because of a lack of relevant good-quality studies.

How, then, do we get the required evidence? The tsunami was a reminder that the divisions within and between nations as well as attempts to close our eyes and borders to problems abroad flounder in the face of the challenges posed by nature [8]. As the world prepares to debate strategies for global equity in health care and the millennium development goals at the G8 summit in July 2005 [9], the lessons learned from the tsunami should not be forgotten. Good-quality systematic reviews form the basis on which interventions should be implemented and on which new interventions should be planned and evaluated [10]. These reviews, however, are only as good as the studies they review. Adequate funding coupled with the necessary volunteers to prepare and maintain systematic reviews of relevant interventions, as well as pragmatic randomised controlled trials to fill the gaps indicated by these reviews, would complete the process initiated by the Cochrane Collaboration, and could well be one of the lasting legacies of the tsunami.

## Abbreviation

PTSD: post-traumatic stress disorder
